# Analysis of DFS70 pattern and impact on ANA screening using a novel HEp-2 ELITE/DFS70 knockout substrate

**DOI:** 10.1007/s13317-017-0091-8

**Published:** 2017-03-17

**Authors:** Kishore Malyavantham, Lakshmanan Suresh

**Affiliations:** Immco Diagnostics, A Trinity Biotech Company, 60 Pineview Drive, Buffalo, New York, 14228 USA

**Keywords:** DFS70, LEDGF, ANAs (antinuclear antibodies), HEp-2 IIF (indirect immunofluorescence)

## Abstract

Indirect immunofluorescence (IIF) using human epithelial cell (HEp-2) substrate is a widely used and the recommended method for screening of antinuclear antibodies (ANA). Dense fine speckled (DFS70) pattern on HEp-2 has been widely reported in various healthy and disease groups. Interpretation of DFS70 pattern can be challenging on a conventional HEp-2 substrate due to its similarity to some of the disease associated patterns. The high prevalence of DFS70 autoantibodies in normal population, lack of association with a particular disease group and a general negative association with systemic and ANA associated autoimmune rheumatic diseases (SARD/AARD) necessitates the confirmation of DFS70 pattern. Results using available commercial assays for confirmation of DFS70 autoantibodies do not always agree with IIF screening results further complicating the lab work flow and ANA algorithms. In this review, we discuss the prevalence of DFS70 antibodies and factors affecting the performance of IIF and DFS70 specific confirmatory assays. Factors that contribute to disagreement between DFS70 suspicion by IIF and confirmatory assays will also be discussed. In addition, we also describe a novel IIF HEp-2 substrate, and its positive impact on DFS70 reporting and ANA screening-confirmation algorithm.

## Introduction

ANAs remain a hallmark of systemic autoimmune diseases. Patterns of ANA observed on HEp-2 cells by IIF provide the clinicians with insight into specificity of autoantibodies present, indications of disease likelihood and further implicate or rule out a clinical suspicion [1]. IIF by HEp-2 is a widely prevalent screening method among the techniques used for the determination of ANA. Despite advances in EIA/ELISA/multiplex methodologies for screening of ANAs, IIF-HEp-2 remains one of the most prevalent methods due to its diagnostic usefulness and cost effectiveness. HEp-2 cells are able to present a variety of autoantigens that result in a multitude of distinct patterns. Though this method has been widely used for more than 50 years, standardization of the quality of HEp-2 substrates (clones, growth phase, fixation method), strength and specificity of FITC-conjugates (fluorescein isothiocyanate), F/P ratio (fluorescein/protein molar ratio), anti-human IgG specificity (Heavy chain/light chain/Fc region), washing technique, buffers, counterstain, microscope setup (excitation light source, use of neutral density filters, narrow/broad band emission filters, quality and specifications of objectives) is lacking. In addition to this, technical expertise and human subjectivity of the readers can impact accurate interpretation of IIF [2]. In an effort to standardize the IIF, International consensus on ANA patterns (ICAP) workshops recommend a consensus nomenclature for the HEp-2 patterns and provide training and description of the nuclear/cytoplasmic/mitosis stage specific patterns on HEp-2 and their antigen/disease associations [3]. The ICAP committee described 28 distinct patterns on HEp-2 and assigned each pattern an AC (Anti-cell) number of 1–28 [3]. DFS70 AC-02 pattern has received the most scrutiny in the field in recent years due to its high rates of prevalence in healthy and ANA positive populations and negative association with SARD/AARD [4, 5].

## DFS70 pattern: background

**Fig. 1:**
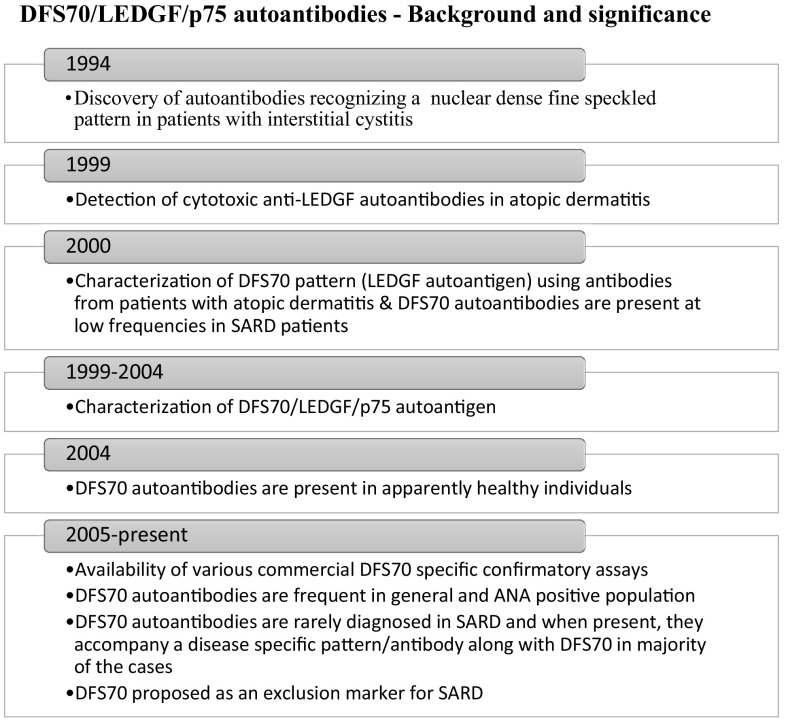
Background, significance and various milestones associated with the discovery and characterization of DFS70 autoantibodies is described

The DFS70 pattern resulting from autoantibodies binding to the ubiquitously expressed protein called lens epithelium derived growth factor (LEDGF) or p75 or psip1 gene product is frequently observed during routine ANA screening by IIF-HEp-2. DFS70 pattern and autoantibodies were originally described by Ochs et al. [8] and were later confirmed in higher frequencies in patients with atopic dermatitis and asthma [6, 7] (Fig. 1). DFS70 is a unique pattern characterized by dense and heterogeneous fine speckled staining of the nucleoplasm in interphase, and speckled staining tightly associated with chromatin during mitosis [7–9]. Independent efforts by various groups have unraveled the identity of the gene encoding this antigen and resulted in characterization of the role of LEDGF/psip1/p75 [7, 10–13]. DFS70/LEDGF/p75 is a ubiquitously expressed growth/transcription factor that localizes to the cell nucleus. The N-terminus has a high affinity for chromatin binding due to which the autoantigen remains tightly associated with chromatin during entire cell cycle [7, 14–16]. Epitope mapping analysis of the DFS70 autoantigen revealed a conformational autoepitope on the C-terminus of the antigen which was responsible for majority of the DFS70 autoantibody binding [17]. DFS70 pattern resulting from LEDGF/p75 gained major attention of the diagnostic field when Watanabe and colleagues reported that 11.6% (64) of the 597 healthy hospital workers in Japan were positive for DFS70 pattern [18]. Role of DFS70/LEDGF/p75 antigen as a transcription factor, cellular co-factor of HIV-1 integration, m-RNA splicing, cell-stress survival factor, its potential interaction with STAT3 in IL-6/STAT3 inflammatory pathway has been reviewed by Casiano and co-workers [19–25]. The mechanism underlying the appearance and the clinical impact of DFS70 antibodies is not yet clear but these have been reported by various groups across the world in both healthy and disease populations. It is still unknown if the DFS70 autoantibodies are natural and protective or pathogenic. Inaccurate interpretation and reporting of AC-02 as one of the disease associated ANA patterns (homogeneous/AC-01, fine speckled/AC-04, speckled/AC-05 or a combination of AC-01/AC-04/AC-05) can lead to unnecessary testing and negatively impact patient care. Due to their high prevalence in ANA screening population and lower association with SARD/AARD, ICAP committee recommends all clinical labs to report the DFS70 pattern [3].

## DFS70 pattern: impact on ANA screening and reporting

**Fig. 2:**
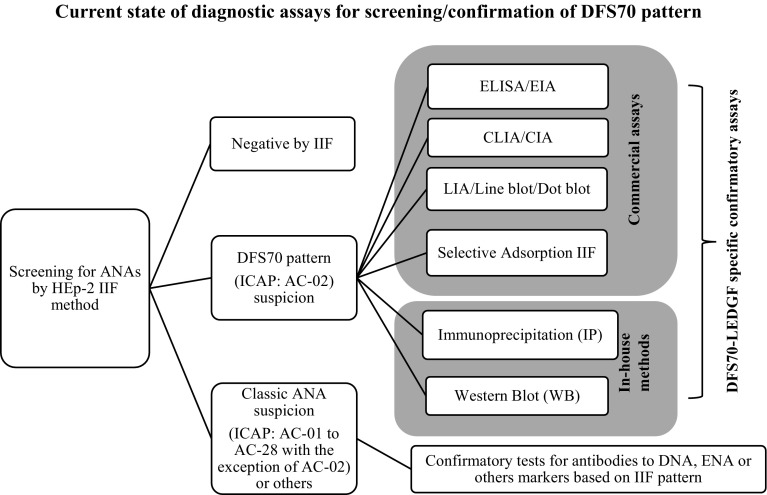
Schematic summarizes the current generation of diagnostic assays used for confirmation of DFS70 pattern and how they are used in the context of ANA screening algorithm

**Fig. 3:**
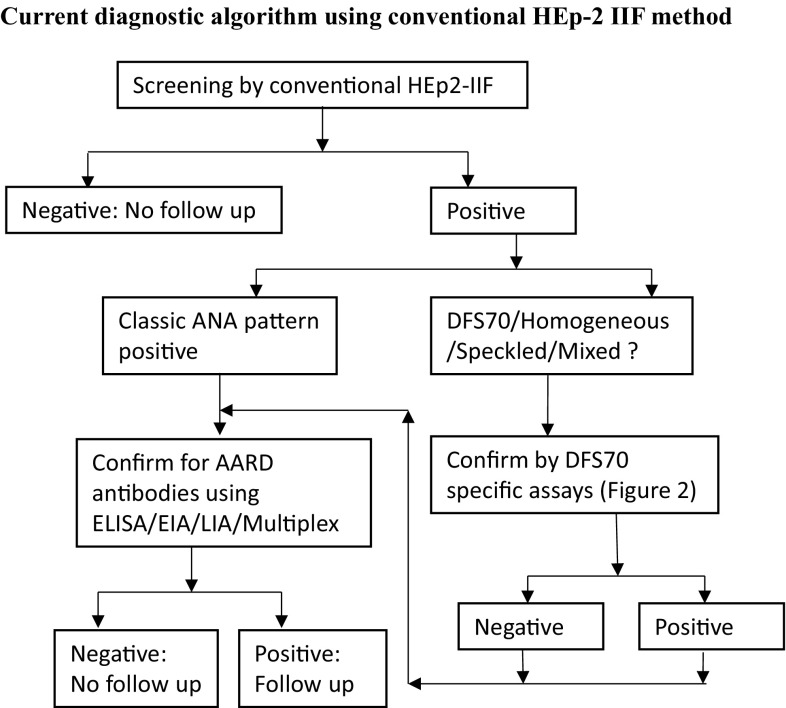
Schematic describes the current diagnostic algorithm for screening ANAs in many labs. IIF using HEp-2 substrates is the first step. Cases that are negative do not need follow up. Positive cases are analyzed for pattern and titer. If disease associated ANA patterns are suspected, respective confirmatory assay/assays are performed. If DFS70/homogenous + speckled mixed pattern is suspected, DFS70 specific confirmatory assays are performed. Both positive and negative DFS70 results on confirmatory assays may warrant additional assays (for autoantibodies towards ENAs, DNA, histone, nucleosome among the others) as presence of disease associated ANAs cannot be ruled out

With the increased demand for ANA testing, many labs have switched to newer solid phase and multiplex methodologies for screening of ANAs [2]. Although these methods are automation friendly and reduce subjectivity in the interpretation of results, they are based on a limited number of purified recombinant/native autoantigens and do not equate in performance to HEp-2 IIF [2]. The American College of Rheumatology(ACR) position statement describes IIF-HEp-2 as the gold standard method for ANA testing [26, 27]. The HEp-2 cell represents at least 100–150 autoantigens in native configuration which provide the unique pattern and titer. This information is of useful value to clinical labs in determining the positive/negative ANA status and selection of appropriate solid phase confirmatory assays [26, 27]. In addition to the ACR, the European autoimmunity standardization initiative group also recognizes the IIF-HEp-2 as reference method despite identifying some of the advantages offered by solid phase/multiplex assays [2]. IIF-HEp-2 is the first step of the routine screening of ANAs and if the IIF result is negative, the samples are not tested further unless there is a strong clinical suspicion (Figs. 2, 3). IIF positive results are analyzed for pattern and titer. Classic disease associated ANA patterns, AC-01 to AC-28 with the exception of AC-02 are further confirmed on appropriate solid phase assays (Figs. 2, 3). Due to the efforts of ICAP and work of experts in the field, clinical laboratories around the world are gaining an understanding of DFS70/AC-02 pattern. DFS70 specific commercial assays are now available for routine use in the form of ELISA/EIA, CLIA/CIA, line blot or dot blot and modified IIF (selective adsorption IIF) procedures (Fig. 2). For research and confirmation of suspected samples, some labs are also using IP (immunoprecipitation) and Western blot assays with cell lysates (HeLa, HEpG2, HEp-2, Jurkat or PC3 cell lines) known to express ample levels of LEDGF/DFS70 protein [25, 28, 29].

## Prevalence of DFS70 autoantibodies

ANA-IIF-HEp-2 is being increasingly requested not only on clinical suspicion of AARD but also for differential diagnosis from AARD. In many clinical laboratories, ANA-IIF referrals come from rheumatologists, hepatologists, neurologists, dermatologists, allergy/immunologists and increasingly from general practitioners to rule out SARD. Based on this trend, the complexity and heterogeneity of ANA screening populations change significantly from one clinical lab to the other. Systematic review for DFS70 autoantibody positivity has been performed by multiple groups [4, 6, 30]. The majority of clinical studies have used IIF-HEp2 for establishing a suspicion of DFS70 pattern and the rates of positivity for DFS70 autoantibodies in each group varied widely between studies [7, 9, 18, 21, 28, 31–44]. DFS70 antibodies have been reported in high titers from cohorts of healthy individuals, blood donors, patients being screened for ANA, patients with various autoimmune disorders and various non-autoimmune disorders including cancers [4, 6, 30]. These studies have shown that DFS70 autoantibodies lack distinct clinical association, with most disease groups, except for certain inflammatory conditions of eyes and skin [4, 6, 7, 18, 30, 44, 45]. The method of screening, selection, and composition of study cohorts may also influence the reported rates of DFS70 autoantibody positivity. A study by Bizzaro et al. [30] using a highly specific commercial DFS70-CLIA method as the first screening step, reported significant variability in DFS70 positivity in clinically defined cases of anti-phospholipid syndrome (60%), Hashimoto’s thyroiditis (47.8%), rheumatoid arthritis (11.1%), Sjogren’s syndrome (4.3%), systemic lupus erythematosus (15.4%), and undifferentiated connective tissue disease (40%) [30]. One hypothesis for this phenomenon is that routine ANA screening by IIF method may not reveal the low levels of DFS70 autoantibodies when disease associated autoantibodies co-exist. Other theories include the challenges associated with setting up an appropriate clinical cut-off value for the confirmatory method.

**Fig. 4:**
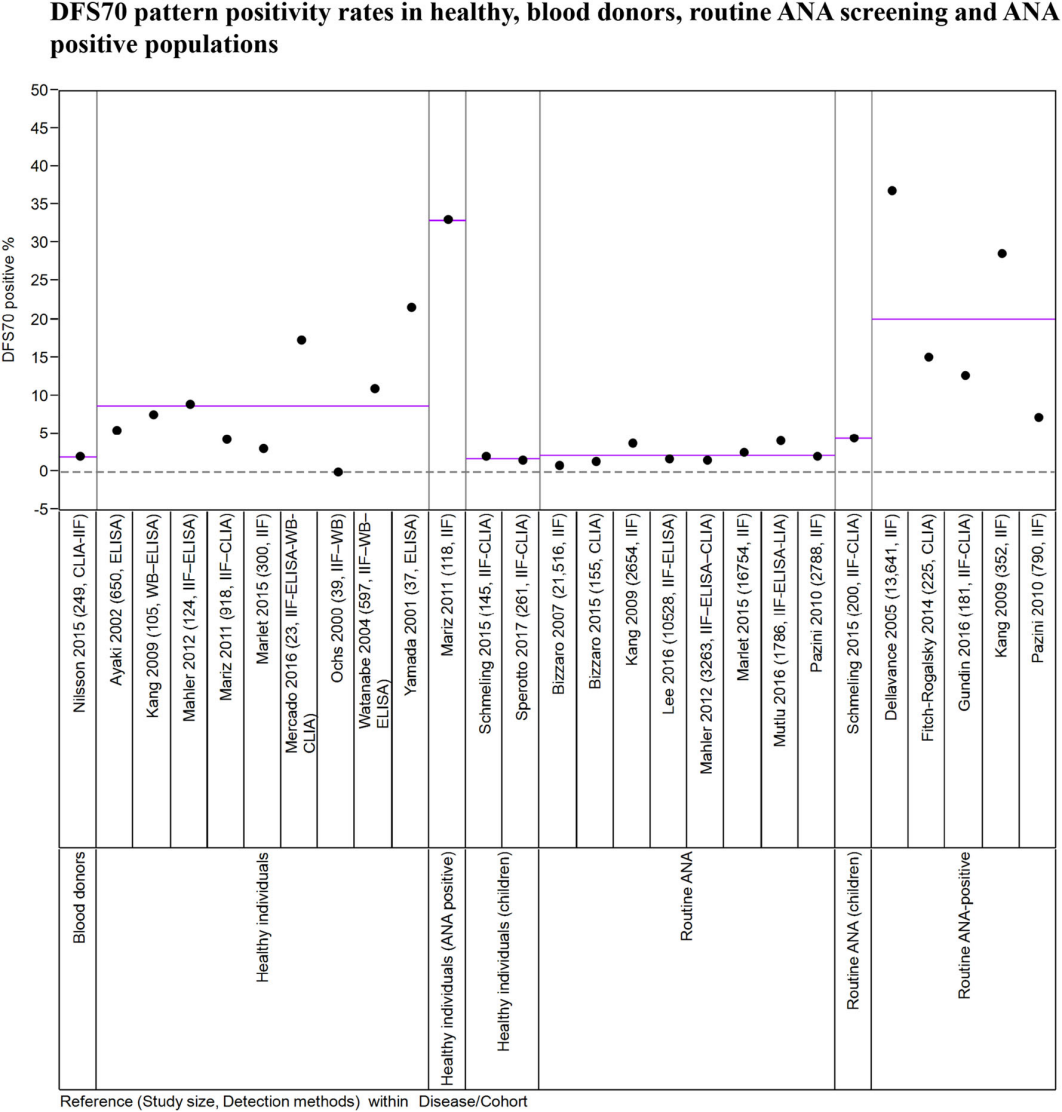
Results from 20 different studies pertaining to reported rates of DFS70 suspicion by IIF/ELISA/CLIA in blood donor, healthy, ANA screening and ANA positive cohorts are depicted. First authors of the study, year of publication is followed by samples size and methods used in* brackets*. Y- axis represents the percent DFS70 positivity reported in each study.* Purple line* represents group mean for each type of cohort

Based on published studies, a great majority of the routine ANA screening population is negative for all ANAs and a large subset of the positive ANA group has DFS70 autoantibodies alone or in combination with other disease associated ANAs. Due to these factors, this review focuses on 20 studies from research group around the world that have reported the frequency of DFS70 suspected cases (Fig. 4). The selected studies provide results from 78,399 cases from various patient cohort types, including blood donors (249), healthy individuals (adult: 2793; pediatric: 406), routine ANA screening populations (adult: 59,444; pediatric: 200), ANA positive healthy individuals (118), and routine ANA screening cohorts with ANA positive status (*n* = 15,189; Fig. 4). The majority of the studies used IIF-HEp-2 as the screening step, with a few using the CLIA or ELISA methods. A detailed review of the results from the selected studies found the rate of DFS70 positivity to be 0–5% in blood donors, healthy children, and in routine ANA screening populations. In contrast, cohorts consisting of healthy individuals that have not been differentiated as pediatric or adults, and ANA positive cases (healthy or routine ANA screening populations) have a higher DFS70 pattern positivity ranging from 0 to 37% (Fig. 4). Group mean for each cohort is indicated by purple lines in Fig. 3 but due to the heterogeneous nature of screening populations, geographic diversity, inter-lab variations in IIF interpretation and accuracy of DFS70 suspicion, the statistics for this data may be of limited value. However, it is clear from the data that DFS70 autoantibodies are highly prevalent in both healthy and disease states where SARD is unlikely. Over and under estimation of DFS70 positivity can have serious impact on patient care and management and clinical labs are obligated to run a number of reflex tests prior to ruling out a suspicion of SARD/AARD (Fig. 3). Many reviews by experts in the field suggested the importance of confirming DFS70 suspicion using specific methods and evaluate its overall impact on ANA screening algorithm and associated costs [1, 4, 6, 30, 34, 39, 46–48]. As per certain studies, approximately a third of the positive ANA cases were positive for DFS70 pattern [9, 33]. Due to these complexities associated with DFS70 autoantibodies, the use of current method of screening significantly increase the number of confirmatory reflex tests run by labs and the financial burden for patients and the system.

## Gap between DFS70 suspicion by IIF-HEp-2 and confirmatory assays

Variations in IIF HEp-2 substrates, screening dilution (1:40/1:80/1:160), inter-observer bias (user training, microscope setup, human subjectivity), FITC-conjugate strength and mixed ANA patterns with/without DFS70 impact IIF reporting. It is also possible that the antibodies that produce DFS70 are very heterogeneous and have increased affinity for full length LEDGF presented in its natural form bound to chromatin and/or other proteins. Wide variability in agreements between IIF suspicion and confirmation by DFS70 specific solid-phase assays have been reported [28, 30, 39, 49]. Confirmatory assay parameters that contribute to this disagreement include differences in antigen selection (full length LEDGF vs. major antigenic region), recombinant expression system used for antigen production (*E. coli* vs. *Baculo virus* system vs. mammalian cells), analytical sensitivity/specificity of the various assay platforms and the established assay cut-off. For a pathologist or a clinical laboratory professional, DFS70 is a distinct pattern that can be differentiated from other similar disease associated patterns. However, depending on the titer levels and presence or absence of other ANA patterns, the interpretation can be challenging [50]. Expert in the field agree that DFS70 autoantibodies can occur in presence of other classic ANAs (SARD/AARD) [37]. Several published studies have suggested the idea of excluding a suspicion of SARD for DFS70 positive subjects but they also highlight the importance of confirming mono-specific or solitary DFS70 antibody positivity [4, 48]. Due to these complexities, the clinical labs run a panel of reflex assays (ENAs, Anti-DNA, Anti-Nucleosome, Anti-Histone assays among the others) for DFS70 pattern suspect cases irrespective of the DFS70 solid phase assay results prior to ruling out the absence of classic ANAs (Fig. 3). Recently proposed selective absorption IIF method (NovaLite, HEp-2 Select, INOVA Diagnostics, USA) uses a high concentration of recombinant truncated LEDGF antigen to cross adsorb DFS70 specific autoantibodies in the sample prior to IIF reaction [51]. Users are expected to implement selective adsorption procedure on DFS70 suspect samples and evaluate the relative reduction in the intensity of DFS70 pattern. While this method attempts to address some of the deficiencies of other solid phase assays, it is an extra IIF assay step and there is a likelihood of incomplete adsorption due to high levels of DFS70 autoantibodies in serum. This possibility reduces the level of confidence for confirming a mono-specific DFS70 reaction and may warrant the use of a second confirmation step for DFS70 and/or multiple confirmatory assays for other ANAs.

## Screening for classic ANAs, detection and confirmation of DFS70 antibodies in one step

**Fig. 5:**
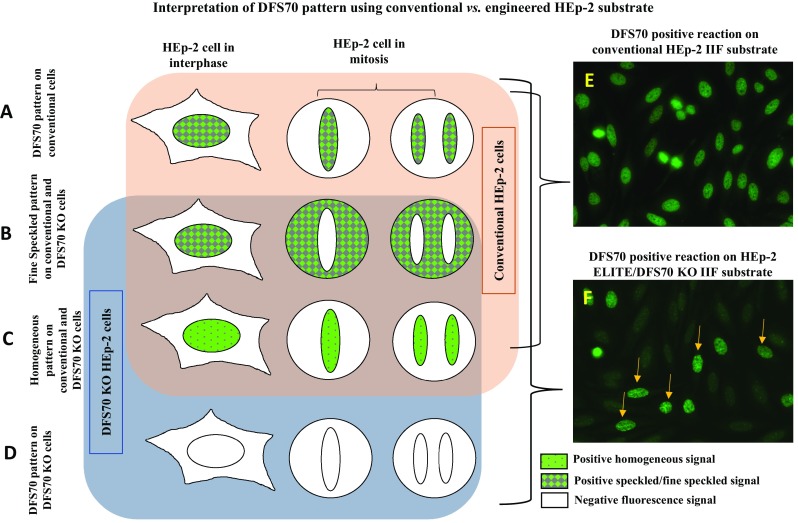
A schematic represents the design of the novel HEp-2 ELITE/DFS70 KO substrate. **a**–**d** Schematics for common patterns (DFS70, homogeneous and speckled) on both interphase and mitotic HEp-2 nuclei in conventional and DFS70 KO cells is described. **e** Example of a DFS70 mono-specific reaction on conventional HEp-2 substrate. **f** Example of a DFS70 mono-specific reaction on HEp-2 ELITE/DFS70 KO substrate is shown. *Arrows* indicate conventional HEp-2 cell nuclei. Negatively stained nuclei are derived from the engineered DFS70 KO cells that do not express LEDGF/psip1/p75 antigen

**Fig. 6:**
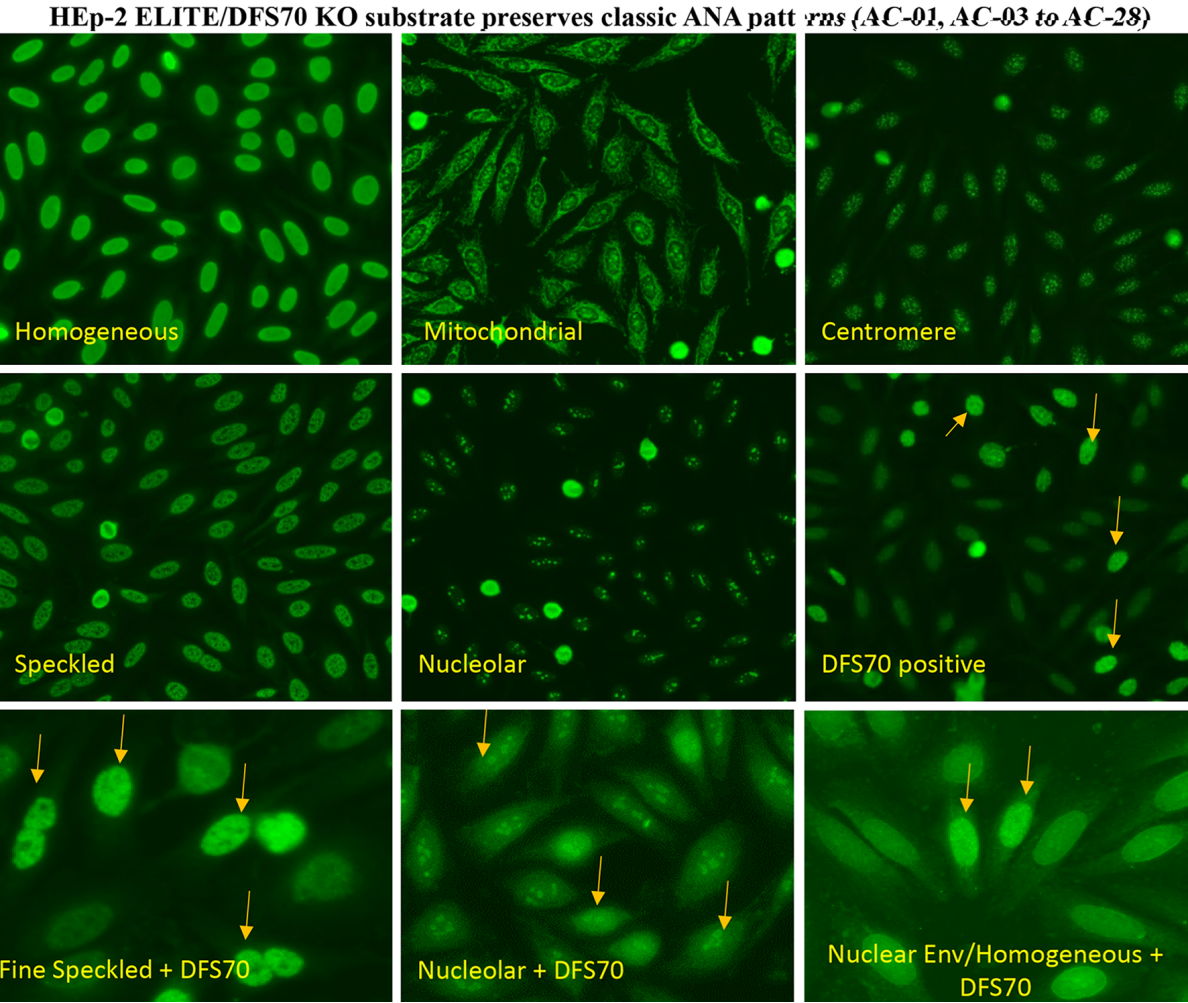
Shows examples of homogeneous, mitochondrial, centromere, speckled, nucleolar and DFS70 (mono-specific) reactions on the new HEp-2 ELITE/DFS70 KO substrates. *Arrows* represent conventional HEp-2 cell nuclei intensely stained with DFS70 reactive serum. For classic ANA patterns both conventional and engineered HEp-2 cells show identical reactions. *Bottom panel* shows examples of mixed patterns revealed on the HEp-2 ELITE DFS70 KO substrates when DFS70 pattern co-exist with another classic ANA pattern. *Arrows* indicate conventional HEp-2 with strong DFS70 pattern. Less intensely labeled DFS70 KO cells are able to reveal fine speckled, nucleolar and nuclear envelope/homogeneous reactions

Here, we introduce a novel HEp-2 IIF substrate (HEp-2 ELITE/DFS70 KO, Immco Diagnostics-Trinity Biotech USA) that presents a mixture of natural HEp-2 cells and genetically engineered HEp-2 cells that do not express DFS70/LEDGF/psip1/p75 antigen (referred to as DFS70 KO cells) in 1:9 ratio on glass slide wells. The new IIF substrate retains all the capabilities of conventional HEp-2 substrates for screening of ANAs and further is able to simultaneously detect and confirm with high confidence both mixed and mono-specific/isolated DFS70 patterns (Fig. 5). Figure 5a–c illustrates how, conventional HEp-2 cells (interphase and mitosis) present classic homogeneous, speckled and DFS70 patterns in natural pattern as expected. Figure 5d shows that the DFS70 KO cells (interphase and mitosis) present only on the novel substrate do not react with DFS70 autoantibodies (Fig. 5d). Therefore, when the substrate is reacted with mono-specific DFS70 sera, a typical pattern with 10% brightly labelled nuclei (derived from conventional HEp-2) and 90% negatively stained nuclei (derived from DFS70 KO cells) is observed. This substrate eliminates the need for evaluation of mitotic pattern to distinguish DFS70 from classic patterns (homogeneous/speckled). Typical reactions obtained using a DFS70 mono-specific sample on conventional HEp-2 IIF substrate (Fig. 5e) and novel HEp-2 ELITE/DFS70 KO substrate (Fig. 5f) emphasize the differences and ease of interpretation. Fine speckled and homogeneous patterns are most frequent in ANA positive cases and are associated with AARD/SARD. These patterns can be distinguished by granular vs. smooth staining of interphase nuclei and negative vs. smooth positive staining of mitotic chromatin. Cases where both speckled and homogeneous patterns co-occur are challenging to distinguish from the DFS70 pattern. HEp-2 ELITE/DFS70 KO substrate is able to present all classic ANA patterns (AC-01 to AC-28 with exception of AC-02) similar to conventional substrates. Representative results from internal studies using HEp-2 ELITE/DFS70 KO substrate produced identical classic ANA patterns when reacted with control sera for respective patterns (Fig. 6). Differential staining was observed only for mono-specific DFS70 (AC-02) pattern and mixed reactions. In case of DFS70 mono-specific reaction, the engineered cells are negative for DFS70 compared to natural HEp-2 cells which show a strong reaction (Fig. 6). In a few cases, the novel substrate revealed classic ANAs that were concealed under the intense DFS70 pattern (Fig. 6: examples of fine speckled, nucleolar and nuclear envelope/homogeneous reactions co-occurring with DFS70 reaction). The new method simplifies the interpretation of DFS70 pattern even in challenging cases presenting low titers of antibodies and mixed patterns.

The preliminary evaluation of HEp-2 ELITE/DFS70 KO substrate was performed by the Laboratory of Clinical Pathology at the San Antonio Hospital located in Tolmezzo, Italy, using a total of 746 cases across five different cohorts. The study included 148 cases suspected of having DFS70 autoantibodies, which were initially identified by conventional HEp-2 IIF (Inova Diagnostics, USA). The other cases evaluated include healthy donors (100), infectious disease positive patients (118), patients diagnosed with an autoimmune disease (138 total; 108 ANA positive and 30 ANA negative), and a routine ANA screening population (242) (unpublished results). The 148 cases suspected of DFS70 pattern by conventional HEp-2 IIF were analyzed using a CLIA assay (QUANTA Flash^®^ DFS70, Inova Diagnostics) and IIF using HEp-2 ELITE/DFS70 KO substrate. The CLIA assay determined 61% (90) of the 148 cases to be positive and 39% (58) as negative. The HEp-2 ELITE/DFS70 KO analysis confirmed 65% (96) of the 148 cases to be positive. New IIF substrate produced a 94% (85) positive agreement with the 90 CLIA positive cases. In addition, the new substrate confirmed approximately a fifth (19%) of the 58 CLIA negative cases to be positive for DFS70 autoantibodies. The new HEp-2 ELITE/DFS KO substrate produced an improved overall sensitivity of 65% compared to 61% obtained with CLIA. The other study cohorts were also tested for DFS70 presence using the HEp-2 ELITE/DFS70 KO substrate. The routine ANA screening population had five cases (2%) identified to be DFS70 positive and the healthy donor population had two cases (2%) as positive. Infectious disease (118) and autoimmune cases (both ANA positive and negative) did not identify any DFS70 positive cases using this improved IIF substrate.

## Conclusion

**Fig. 7:**
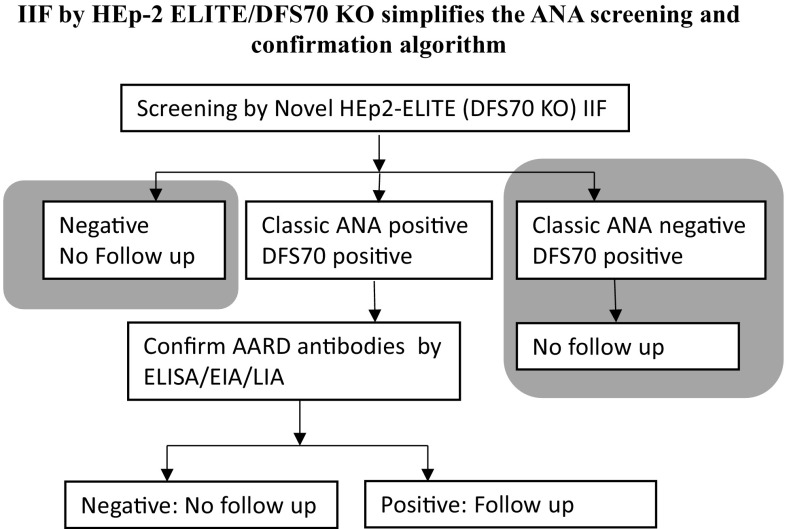
HEp-2 ELITE/DFS70 KO substrate as the first step IIF screening method simplifies the ANA screening and confirmation algorithm. Ability to detect and confirm DFS70 mono-specific reactions while simultaneously screening for disease associated ANAs reduces the number of confirmatory assays. *Grey boxes* represent a large subset of ANA screening population who may not need a follow up in absence of a strong clinical suspicion for AARD/SARD. Using the new substrate, these cases do not need additional testing. Classic ANA positive cases with or without DFS70 pattern will be confirmed by appropriate solid phase confirmatory assays

DFS70 autoantibodies have been reported by numerous groups not only in various autoimmune and non-autoimmune disease states but also in healthy population. DFS70 autoantibodies present a unique interpretation challenge for clinical labs that use the recommended HEp-2 IIF for screening of ANAs. Currently available commercial assays for the confirmation of DFS70 autoantibodies do not always agree with DFS70 suspicion by IIF. Over and under estimation of DFS70 pattern using conventional IIF complicates the ANA screening work flow by increasing the number of reflex tests which further increases the cost of implementing the diagnostic algorithm (Fig. 3). The novel HEp-2 ELITE/DFS70 KO substrate presented here simplifies the interpretation of DFS70 pattern (Fig. 5) and improves the overall accuracy of the ANA screening algorithm by revealing classic ANA reactions masked by DFS70. This new substrate can screen and confirm mono-specific or isolated DFS70 positive cases in one step while adhering to the standard IIF methodology and not compromising on the abilities of a conventional HEp-2 IIF method (Fig. 6). A major subset of the routine ANA screening population consists of ANA negative and DFS70 positive cases which if confirmed with confidence do not need a clinical follow-up (Fig. 7). The current generation of DFS70 specific confirmatory assays neither provide high levels of agreement with IIF results nor are able to confirm the mono-specific/isolated DFS70 positivity, thereby complicating the ANA screening and confirmation algorithm (Fig. 3). Therefore, to eliminate suspicion of SARD/AARD, clinical labs rely on a large panel of ANA specific assays even in cases of DFS70 suspicion (Fig. 3). Implementation of the newly described HEp-2 ELITE/DFS70 KO substrate as the first step IIF, significantly improves and simplifies the ANA screening and confirmation algorithm (Fig. 6). The new HEp-2 ELITE/DFS70 KO substrate overcomes the limitations associated with accurate interpretation of DFS70 pattern and increases the overall accuracy of the HEp-2 IIF method for screening of ANAs.

## Abbreviations

ANA: Antinuclear antibody
CLIA: Chemiluminescence immunoassay
DFS70: Dense fine speckled 70
EIA/ELISA: Enzyme-linked immunosorbent assay
ICAP: International consensus on ANA pattern
IIF: Indirect immunofluorescence
LEDGF: Lens epithelium derived growth factor
AARD: ANA associated rheumatic diseases
SARD: Systemic autoimmune rheumatic diseases
